# Influence of Framing Effect on Consumers’ Purchase Intention of Artificial Meat—Based on Empirical Analysis of Consumers in Seven Cities

**DOI:** 10.3389/fpsyg.2022.911462

**Published:** 2022-06-02

**Authors:** Lijie Shan, Xinli Jiao, Linhai Wu, Yingcheng Shao, Lingling Xu

**Affiliations:** ^1^Institute for Food Safety Risk Management, Wuxi, China; ^2^School of Business, Jiangnan University, Wuxi, China; ^3^School of Business, Sichuan University, Chengdu, China

**Keywords:** artificial meat, purchase intention, framing effect, product knowledge, health motivation

## Abstract

Artificial meat is a type of food that has emerged in recent years. It is similar in shape, color, and taste to meat. Its market scale is developing rapidly, and its future development prospect is bright. To explore Chinese consumers’ purchasing intention regarding artificial meat products, this study used the framing effect theory to analyze the differences in consumers’ purchasing intentions under different information frames based on the survey data of 6,906 consumers from seven cities in China. Hierarchical regression and variance analysis explored the moderating effects of consumers’ product knowledge level and health motivation on the frame effect. The results show that consumers’ purchase intention under the positive information frame is significantly higher than that under the negative information frame. Consumers with higher product knowledge levels have higher purchase intention under the positive information frame, whereas consumers with lower health motivation have lower purchase intention under the two information frames. The government and relevant enterprises should focus on promoting positive information about artificial meat products, improving consumers’ cognition level of artificial meat products, guiding consumers to form a scientific diet concept to enhance their purchase intention of artificial meat products, and promoting the healthy development of the artificial meat industry.

## Introduction

With the growth of population, economic development, and deepening urbanization, global meat consumption continues to increase, especially in developing countries, led by China and India. Consequently, the demand for meat is rising rapidly, resulting in a widening gap between meat demand and supply (Tobler et al., 2011). According to the China Agricultural Outlook Report (2020–2029) released by the Ministry of Agriculture and Rural Affairs, the total meat consumption in China will increase by 20.7% in the next decade. The gap between the supply and demand of meat products in China is expected to reach 38.4 million tons by 2030 (Xin et al., 2018a). The continuous increase in meat consumption poses potential threats to the sustainable development of humans, the environment, and animal welfare. For example, the explosive growth of feed and grain demand has adversely impacted food security, and animal husbandry has brought about environmental pollution, such as greenhouse gases, causing approximately 22% of all foodborne diseases (FAO, 2009; Gerber et al., 2013; Bonny et al., 2015; Xin et al., 2018b), causing significant economic losses (Wang et al., 2020). In this context, the market size of meat substitutes, such as artificial meat has grown steadily, and currently, there are two main types of artificial meat. One uses plant proteins and other plant components to synthesize artificial plant meat with characteristics similar to meat. The other is cultured meat that uses animal stem cells grown in a medium under certain conditions (Zhang and Tu, 2020). Artificial meat has greater advantages in promoting environmental protection, physical health, and increasing animal welfare compared to real meat. It is also conducive to reducing feed grain and ensuring China’s food security (Tuomisto and de Mattos, 2011; Bhat et al., 2015; Mattick et al., 2015). Data show compared to conventional meat production, artificial meat may reduce energy consumption and land usage by 99%, water usage by 90%, and energy consumption by 40% (Tuomisto and de Mattos, 2011).

As artificial meat is an emerging food, relevant studies by scholars are still relatively rare and mainly focus on processing technology and other aspects. The few consumer studies are mainly aimed at consumers in Western countries, such as Britain and the United States, focusing on factors affecting consumers’ purchase intention (Hoek et al., 2011; Wilks and Phillips, 2017). Only Zhang et al. (2020) researched Chinese consumers’ acceptance of artificial meat. For example, consumers’ individual characteristics, attitude to artificial meat, and external information will affect consumers’ purchase intention to artificial meat (Verbeke et al., 2015b; Wilks and Phillips, 2017; Slade, 2018). However, consumers are not always completely rational when facing external information but tend to be bounded rationality (Simon, 1955). Different ways of describing the same information may produce a framing effect, leading to certain differences in consumers’ cognition and purchase intentions (Tversky and Kahneman, 1981). For example, Levin and Gaeth (1988) presented an advertisement for ground beef to two groups: one was framed as “75% lean” and the other as “25% fat.” Participants responded more favorably toward the beef when it was described as 75% lean. In the case of artificial meat, an emerging food product, how can information be presented to consumers to maximize consumers’ perception and purchase intention of artificial meat? This issue needs to be further explored. Based on this, this study attempts to apply the frame-effect theory, taking consumers in seven first-tier or quasi-first-tier cities, such as Shanghai, Beijing, and Guangzhou, as research objects, using variance analysis, hierarchical regression, and other methods to explore the influence of different information frames on consumers’ purchase intention of artificial meat products, and the moderating effect of consumers’ product knowledge level and health motivation on the framing effect. To improve consumers’ awareness of artificial meat, improve consumers’ purchase intention, and promote the development of the artificial meat industry to provide reference and countermeasures.

## Literature Review and Research Hypotheses

According to the “rational economic man” hypothesis in western economics, individual behavioral decisions always follow the principle of maximum utility and have a certain inherent constancy, which is less affected by external factors (Chen, 2000). However, with the development of behavioral economics, further study has found that the behavioral paradox of “rational economic man” in real situations has occurred from time to time (He, 2005). The prospect theory proposed by Tversky and Kahneman (1979) is based on the theory of bounded rationality, which believes that the bounded rationality of individuals affects the results of decision-making (Li et al., 2019; Shan et al., 2019). In other words, consumers’ decisions do not always follow the principle of rationality but may be made based on external information and consumers’ experience in real situations, and this decision may not be optimal. In the process of consumers purchasing products, due to the limitations of their information processing capabilities, the same information is described in different ways, which may change the consumer’s cognitive reference point, affecting their behavior or willingness. When Tversky and Kahneman (1981) studied the “Asian disease problem,” they found that the same information described in different ways would lead to changes in individual behavioral decisions and thus produce the framing effect.

Levin et al. (1998) conducted a meta-analysis of frame effects in different fields and divided the frame effects into three types: risk frame effects, attribute frame effects, and target frame effects. Among them, the goal-framing effect is more widely applied in studying consumers’ behavioral intention, and more emphasis is placed on the consequences of behavioral choice (Jin and Zhang, 2015; Li et al., 2018). According to the differences in the description of the behavioral consequences, the target frame can be divided into a positive information frame and a negative information frame, which refers to the semantic descriptions of the possible positive effects of behavioral choices, such as doctors telling people about the benefits of colorectal cancer screening; the negative information frame refers to the semantic description of the possible adverse effects of behavioral choices, such as the physician’s introduction of the risks people may face if they do not receive colorectal cancer screening (Todd et al., 2018; Dai et al., 2020).

### Framework Effect and Consumer Purchase Intention

The framing effect exists in many fields, such as health examination, advertising and marketing, and financial investment (Liu et al., 2010; Lin and Yeh, 2017; Wang et al., 2020). In terms of consumer willingness and behavior, existing studies show that consumers’ purchase intentions or behavior are generally affected by the framing effect (Velde et al., 2010; Dai et al., 2015; Li et al., 2016), but the type of information framework that has a more significant impact on consumers’ purchase intentions? There is still controversy on this issue. Velde et al. (2010) believe that a positive information frame can significantly improve the effectiveness of consumers’ perceived information; thus, consumers have a higher purchase intention under the positive information frame. Wu and Cheng (2011) and Yang et al. (2018) also held a similar view; that is, message framing affects consumers’ purchase intention, and consumers have higher purchase intention under the positive information framework. However, Meyerowitz and Chaiken (1987) found that consumers are more sensitive to negative information in the information editing process and are more susceptible to negative information. Therefore, negative message framing has a more significant impact on consumers’ purchase intention. The study of Sheng et al. (2019) also found that emphasizing the negative consequences caused by not buying green products may stimulate consumers’ fear, thus improving consumers’ purchase intention. In terms of artificial meat products, Verbeke et al. (2015a) conducted a study on Belgian consumers and found that consumers who knew more about the positive information of artificial meat had higher acceptance and purchase intention than those who did not. Accordingly, this article puts forward the following assumptions:

*H1*: Framing effect affects consumers’ purchase intention toward artificial meat, and respondents facing positive message framing will be more likely to purchase artificial meat than those facing a negative message framing.

### Moderating Effects of Consumer Knowledge Level and Health Motivation on Framing Effect

Consumers’ product knowledge level and motivation affect the extent and level of information processing (Petty and Cacioppo, 1986; Frias et al., 2008). When individuals have a higher level of product knowledge and motivation, they are more capable and willing to carry out a rational and comprehensive in-depth analysis of the received information. However, when individuals lack sufficient knowledge and motivation, they tend to make simple inferences or judgments based on their own experience and situation (Lai and Tang, 2017).

Product Knowledge Level. Consumers with different levels of product knowledge recognize and edit different message frames differently, resulting in framing effect and forming different levels of purchase intention (Costa-Font and Gil, 2009; Liu, 2019). Consumer product knowledge level plays a significant moderating role in the influence of the information frame on purchase intention (Yu and Mao, 2019). Consumers with a higher level of product knowledge can actively compare the information of different frames with their professional knowledge and analyze the information more rationally, thus weakening the framing effect. Consumers with a low level of product knowledge mainly make simple inferences or judgments based on incomplete experience, so they are more susceptible to the framing effect (Kinder and Sanders, 1990). Moreover, consumers’ purchase intention under positive message framing is significantly higher than that under negative message framing (Jin and Han, 2014). This leads to the following hypotheses:

*H2*: Consumer’s artificial meat knowledge level has a significant adjustment of the framing effect, affecting the purchase intention.

Motivation. Individual motivations affect consumers’ level of information processing and processing (Xie and Wang, 2003), resulting in a framing effect, leading to differences in consumer purchase intentions with different motivation levels. Suri et al. (2003), Xie and Wang (2003), and Chang and Wu (2015) found that individual motivations of different levels all have a significant moderating effect on the framing effect. Consumers with lower motivation levels have lower purchase intentions under both information frames, whereas consumers with higher motivation levels have significantly different purchase intentions under different information frames. As an emerging food, consumers’ purchasing motivation is mainly reflected in environmental protection and health. That is, buying artificial meat is beneficial to protect the environment, promote sustainable development, and reduce potential disease risks (Yuan et al., 2021). The “Survey Report on Environmental Awareness of Chinese Urban Residents” released by Shanghai Jiao Tong University in 2019 shows that although the environmental awareness of Chinese urban residents has improved compared with the past, their environmental protection willingness and behavioral tendencies have not yet been fully translated into practical actions. Therefore, this article mainly explores the moderating effect of consumers’ health motivation on the framing effect. Consumers’ health motivation is closely related to their attitudes toward healthy eating. Consumers who pay more attention to dietary health have correspondingly higher health motivations, and those who pay less attention to dietary health have correspondingly weaker health motivations (Xu and Zhao, 2010). Therefore, this study adopts consumers’ attention to a healthy diet to measure their health motivation and puts forward the following assumptions:

*H3*: Consumers’ health motives have a significant moderating effect on the frame effect, which in turn affects consumers’ purchase intentions.

## Materials and Methods

### Experimental Design

At present, cell culture meat is expensive and has not been listed in China, whereas plant-based meat has been used for large-scale production and market operations (Zhao et al., 2019). Therefore, this questionnaire survey takes artificial plant-meat products as the research object. Based on the design ideas of Roininen et al. (1999), Wu and Cheng (2011), Yin et al. (2014), Wilks and Phillips (2017), Konuk (2018), Duan et al. (2019), and Gómez-Luciano et al. (2019), two versions of the questionnaire, A and B, were designed. The content of the questionnaire consists of three parts. The first part investigates consumers’ eating habits, health motivations, and consumers’ knowledge of artificial meat products and trust levels. The second part provides the interviewees with relevant information about artificial meat, after reading the product information, interviewees answer questions related to purchasing intentions. The third part investigates consumers’ characteristics. The contents of the questionnaires in the A and B versions—which are, respectively, a negative information frame message and a positive information frame message—are only different in the second part. Relevant questions were asked in the form of statements. A 5-point Likert scale ranging from 1 (“strongly disagree”) to 5 (“strongly agree”) is used to express respondents’ views, with higher levels indicating the corresponding level of knowledge, health motivation, and purchase intentions. Among them, the knowledge level scale includes two items (Do you know about artificial plant meat? Do you understand the difference between artificial plant meat and poultry meat?). The health motivation scale includes four items (1. For me, the food I eat every day contains a lot of vitamins and minerals are very important. 2. For me, it is very important that the food I eat every day can strengthen my health. 3. I attach great importance to whether the food I eat on weekdays is nutritious. 4. It is important to me that my daily diet is high in protein.). The purchase intention scale includes three items (1. Would you like to try plant-based meat? 2. Would you like to buy plant-based meat instead of poultry meat? 3. Would you recommend buying plant-based meat to your family and friends?), and consumer trust measures consumers’ trust in the quality of artificial meat products.

Based on the research of Yu and Mao (2019) and Shan et al. (2020), this study adopted negatively and positively framed messages for artificial meat advertisements. Questionnaire A negatively framed artificial meat lettuce by stating the following:

Plant-based meat is a type of “artificial meat.” It is a meat substitute with animal meat fiber structure and taste produced by a special process with vegetable protein, such as soy protein as the main raw material and appropriate auxiliary materials. With the gradual increase in *per capita* meat consumption, humans may face many potential health threats and pose many environmental hazards and problems if artificial meat is not purchased or consumed. Poultry meat is mainly produced by intensive methods. The breeding environment is crowded and harsh. Antibiotics and drugs may be used in the breeding process. Long-term consumption of large quantities of poultry meat may be detrimental to health. Furthermore, livestock farming consumes increasing amounts of water, energy, food, and land resources and is responsible for higher greenhouse gas emissions than the transportation industry, causing serious environmental hazards.

For positive framing, it described the same product using the following phrasing:

Plant-based meat is a type of “artificial meat.” It is a meat substitute with animal meat fiber structure and taste produced by a special process with vegetable protein, such as soy protein, as the main raw material and appropriate auxiliary materials. Artificial meat has a protein content of greater than 50% and zero cholesterol, low in fat, and high in protein. The purchase and consumption of artificial meat will help reduce health risks due to excessive meat intake, such as cardiovascular disease. Moreover, the large-scale production and consumption of artificial meat will greatly reduce the consumption of natural resources, such as arable land area, food, and water, by animal husbandry and reduce greenhouse gas emissions to achieve the sustainable development of human society and the natural environment.

### Experimental Organization

At present, the market coverage of artificial meat products is limited, mainly in chain restaurants in cities with higher levels of economic development, and sold as finished products, such as burgers. Therefore, to launch consumer surveys this research group selected seven first-tier, new first-tier, or provincial capitals, Beijing, Shanghai, Guangzhou, Shenzhen, Zhengzhou, Chongqing, and Xi’an. The above seven cities have a high level of economic development, and consumers are more open-minded and more likely to accept new things. Moreover, all the above cities have restaurants selling artificial meat products. For example, Chongqing City has launched an “artificial meat hot pot,” and Zhengzhou City has more than 20 restaurants selling artificial meat products. In addition, the above seven cities are from East China, North China, South China, Southwest, and Northwest China, and the geographical differences, to a certain extent, can reflect the dietary characteristics and preferences of consumers in different regions of China.

A questionnaire survey was conducted by consulting professional consulting companies in the form of an online survey due to the large sample size. The questionnaire company conducted the formal survey in the above cities, and simultaneously, a regional screening question was added to the questionnaire to conduct a second screening of the survey group. In addition, an IP address filter was set when questionnaires were placed to ensure the uniqueness of each questionnaire. The response time of each questionnaire was restricted. Questionnaires with a response time of less than two and a half minutes were judged as unqualified. Monetary compensation was provided after the questionnaire was answered to encourage consumers to answer the questionnaire carefully. The survey was conducted from April 5 to May 30, 2020, and two versions of questionnaires, A and B, were placed in the above seven cities in a ratio of 1:1. 6906 valid questionnaires were returned, including 3,452 questionnaires in version A and 3,454 in version B.

Of the total respondents, 50.61% were female, which was slightly higher than males (49.39%). The majority of respondents were young and middle-aged, with 77.41% aged 26–45 and 66.8% married. The overall education level of the respondents was relatively high, and 79.54% had a high school or bachelor’s degree. In addition, the income distribution of the respondents was uneven. The low-income group with an annual income of less than 2,790 USD and the high-income group with an annual income of more than 22,320 USD account for a relatively low proportion of respondents, and 76.15% of the respondents had an income between 5,580 USD and 22,320 USD. The occupational distribution of the interviewees was widely distributed, and more than one-third of the respondents were general employees of enterprises and institutions (Table 1).

**Table 1:** Demographic characteristic of participants.

Demographics	Classification	Negative information framework (Questionnaire A)	Positive information framework (Questionnaire B)	Ratio (%)
Gender	Male	1,676	1,735	49.39%
	Female	1,776	1,719	50.61%
Age	18–25	677	637	19.03%
	26–35	1,762	1,828	51.98%
	36–45	878	878	25.43%
	46–55	126	100	3.27%
	56 and above	9	11	0.29%
Education	Less than junior college	91	86	2.56%
	Junior college	633	603	17.90%
	Junior college	956	888	26.70%
	Undergraduate course	1,651	1,739	49.09%
	Postgraduate and above	121	138	3.75%
Marital status	Married	2,230	2,300	65.60%
	Unmarried	1,222	1,154	34.40%
Income	2,790 USD and less	271	231	7.27%
	2,790–5,580 USD	363	324	9.95%
	5,580–9,300 USD	753	731	21.49%
	9,300–14,880 USD	1,144	1,160	33.36%
	14,880–22,320 USD	681	790	21.30%
	More than 22,320 USD	240	218	6.63%
Profession	Student	409	396	11.66%
	Managers	535	563	15.90%
	Ordinary staff	1,282	1,249	36.64%
	Professionals	423	486	13.16%
	Migrant workers	380	338	10.40%
	Self-employed/contractor	333	340	9.75%
	Farmers	37	43	1.16%
	Others	53	39	1.33%

### Validity and Reliability

This study used SPSS 20.0 and SPSSAU to test the reliability and validity of the questionnaires.

The exploratory factor analysis results in Table 2 show that the Kaiser–Meyer–Olkin (KMO) values of all scales were greater than 0.5, and the Bartlett sphericity test results were significant, indicating that the questionnaire is suitable for further reliability and validity test analyses. Table 2 shows the reliability of each item using Cronbach’s alpha. The values are 0.797 (knowledge), 0.783 (health motivation), and 0.913 (purchase intention). These reliability coefficients are higher than the critical value of 0.60, suggesting high internal reliability (Ma and Pan, 2000).

**Table 2:** Validity and reliability of study variables.

Variables	Latent variables	Factor loading	KMO	Bartlett	Cronbach’s alpha	CR	AVE
Knowledge	KL1	0.984	0.500	3475.949^***^	0.797	0.834	0.723
	KL2	0.673					
Health motivation	HM1	0.731	0.787	6361.489^***^	0.783	0.783	0.475
	HM2	0.681					
	HM3	0.695					
	HM4	0.649					
Purchase intention	PI1	0.871	0.758	12493.248^***^	0.913	0.914	0.779
	PI2	0.871					
	PI3	0.903					

*** Indicates that the significance level of Bartlett’s sphericity test is less than 0.01.

Based on the reliability test of the questionnaire, confirmatory factor analysis is used to test the convergent validity and discriminative validity. Convergent validity generally has three measures: standardized factor load, combined reliability (CR), and average extraction variance (AVE). When the standardized factor load is greater than 0.5, the CR is greater than 0.7, and the AVE value is greater than 0.5, the questionnaire is said to have good convergent validity (Wu, 2010). The factor loading coefficients of all variables in this questionnaire are greater than 0.5, the CR values are greater than 0.7, the AVE values of the average extraction variance of knowledge level and purchase intention are greater than 0.5, and the AVE of health motivation is 0.475, which is also close to 0.5, indicating that the scale has good convergent validity. Discriminant validity, showing the degree of constructs measured using different methods, is distinguishable. One principle for discriminant validity is that the correlation coefficient between one construct and the others should be less than the square root of the AVE for each variable. The diagonal of Table 3 presents the AVE square roots, all of which are greater than the correlation coefficient, indicating favorable discriminant validity.

**Table 3:** Average variance extracted and correlation of constructs.

	Knowledge	Health motivation	Purchase intention
Knowledge	0.851^*^		
Health motivation	0.067	0.689^*^	
Purchase intention	0.179	0.195	0.883^*^

* The diagonal row numbers are square roots of the AVE. Off-diagonal numbers are the correlations among variables.

The reliability and validity test results indicate that the questionnaire data have good reliability and validity, which meets the needs of this study.

### Manipulation Test of Information Framework

The questionnaires in the A and B versions are designed with related questions to test the manipulation effect of the information framework. After the interviewees read the negative frame information of questionnaire A and answered the question, “Does the above information introduce us to the health threats and environmental hazards that may be caused by not buying artificial meat?” 77.75% of the interviewees chose “Yes,” 22.25% of the respondents chose “No,” the chi-square value is 1063.458, the degree of freedom is 1, and the significance level value of *p* is less than 0.01, indicating that most of the respondents have accepted the information provided by the negative information framework. Respondents read the positive framework information of questionnaire B and answered the question, “Does the above information introduce us to the health benefits and environmental protection effects of buying artificial meat?” 96.76% of the respondents chose “Yes,” 3.24% of the respondents chose “No,” the chi-square value is 3020.527, the degree of freedom is 1, and the significance level value of *p* is less than 0.01, indicating that most of the respondents have accepted the information provided by the positive information framework.

Thus, most respondents could effectively identify the information provided by positive and negative information frames, and the information frames were designed with reasonable content.

## Results and Discussion

We used ANOVA to test the framing effect on consumers’ purchase intentions toward plant-based meat products. Further, we examined the role of consumers’ knowledge and health motivation using ANOVA and hierarchical multiple regression (HMR).

### Main Effect of Framing

The results of the one-way ANOVA showed that the value of the F-statistic for consumer purchase intention under different information frameworks was 90.164 with a significance level value of *p* less than 0.01, indicating that information frameworks have a significant effect on consumer purchase intention. Further analysis revealed that the mean value of consumers’ purchase intention in the positive information frame was M positive information frame = 3.46, and the mean value of consumers’ purchase intention under the negative information frame was M negative information frame = 3.21, M_positive information frame_ > M_negative information frame_, indicating that consumers’ purchase intention for plant-based meat was higher under the positive information frame, and Hypothesis 1 was verified. This finding is contrary to the findings of Chang and Wu (2015) and Shan et al. (2020). Possible reasons for this are, first, that plant-based meat, the subject of this study, is an emerging food and consumers have limited knowledge about it, and the positive information frame provides respondents with more positive information about plant-based meat, which makes respondents form positive evaluations of artificial meat and is more likely to arouse their curiosity and stimulate consumers’ willingness to buy. Second, according to prospect theory, there may also be differences in the effects of positive and negative information frames for behaviors with different risk probabilities (Rothman and Salovey, 1997). The impact of positive information framing is more significant when individuals perceive a lower risk of engaging in the behavior (e.g., health-related disease prevention behaviors; Luo et al., 2013). In the present study, the purchase and consumption of plant-based meat products by consumers was relatively low-risk. Therefore, consumers’ intention to purchase artificial meat was higher under the positive information framework.

### Role of Product Knowledge in Framing Effect

When the moderating variable is a continuous variable, the use of hierarchical regression models allows for a more intuitive and precise observation of whether there is a moderating effect and whether the moderating variable is significant (Wen et al., 2005). Based on this, this study uses a hierarchical regression model to analyze the moderating effect of the level of consumer knowledge of artificial meat on the information framework. The results of the hierarchical regression analysis are shown in Table 4 using the information frame and consumer knowledge level and the interaction term between the two as independent variables and purchase intention as the dependent variable.

**Table 4:** Moderating effect of knowledge level on frame effect.

Variables	Purchase intention					
	Model 1		Model 2		Model 3	
	*β*	*t*	*β*	*t*	*β*	*t*
Constant term	0.888^**^	9.764	0.907^**^	9.923	0.901^**^	9.862
Gender	0.172^**^	8.491	0.175^**^	8.615	0.177^**^	8.69
Age	−0.01	−0.611	−0.012	−0.705	−0.011	−0.684
Education	0.069^**^	5.758	0.068^**^	5.659	0.068^**^	5.671
Marital status	−0.1^**^	−3.741	−0.096^**^	−3.579	−0.095^**^	−3.542
Income	0.041^**^	4.436	0.04^**^	4.25	0.039^**^	4.24
Trust	0.616^**^	57.378	0.611^**^	55.597	0.611^**^	55.64
Message frame (M)			0.21^**^	10.428	0.21^**^	10.437
Knowledge (K)			0.027^*^	2.047	0.026^*^	1.973
M^*^K					−0.058^*^	−2.309
*F*	569.213^**^		498.849^**^		444.333^**^	
*R* ^2^	0.398		0.399		0.399	
∆F	569.213^**^		4.189^*^		5.332^*^	
∆R^2^	0.198		0		0.001	

^*^p < 0.05; ^**^p < 0.01.

**Figure 1:**
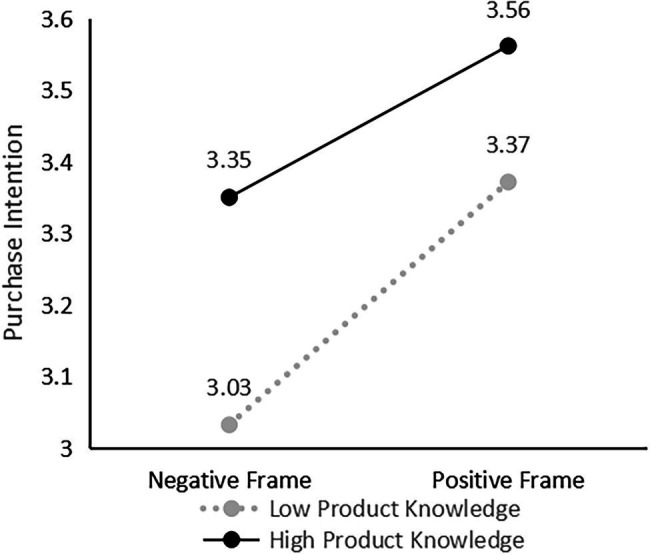
Moderating effect of knowledge level on framing effect.

The results (Table 4) showed that the interaction term of information frame and knowledge level had a significant effect on consumers’ intention to purchase artificial meat at the 5% level, indicating that the level of knowledge of artificial meat acquired by consumers plays a significant moderating role in the effect of information frame on purchase intention. Hypothesis 2 was verified, a finding consistent with the findings of Yu and Mao (2019). Consumers’ knowledge level is divided into high level and low level groups according to the median. The simple slope method is used to analyze the moderating effect of knowledge level on the framing effect. The results are shown in Figure 1. When categorized based on their knowledge level according to their survey scores, more knowledgeable consumers were less likely to change their attitudes or purchase intentions based on the message frame. A possible reason is that when consumers have more knowledge about artificial meat, they can more accurately identify and understand the information they receive and make judgments in combination with the knowledge they already have and are less likely to be influenced by the framing effect. When consumers have little knowledge about artificial meat, they will rely more on the external information they receive and make decisions based on their intuition and are more likely to be affected by the framing effect.

### Role of Health Motivation in Framing Effect

**Figure 2:**
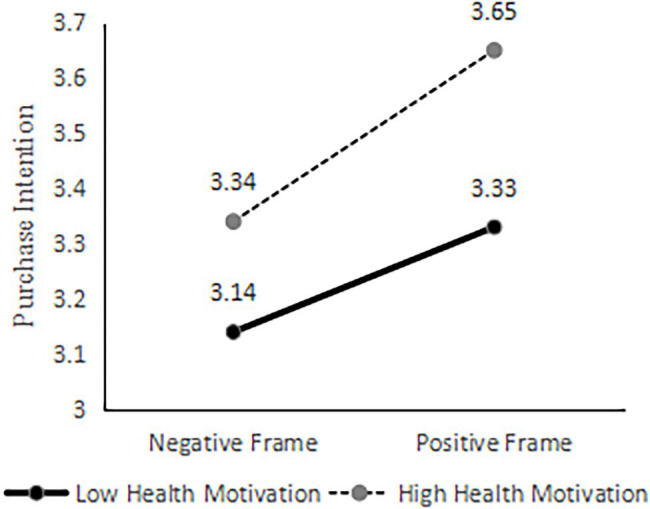
Moderating effect of health motivation level on framing effect.

A two-factor ANOVA with purchase intention as the dependent variable and information frame and consumer health motivation as independent variables showed that the F-statistic value of the interaction term between information frame and health motivation was 5.000 with a value of *p* of 0.025, indicating that consumer health motivation plays a significant moderating role in the effect of frame effect on purchase intention, and Hypothesis 3 was verified (Table 5). Consumers’ health motivation is divided into high level and low level groups according to the median. And then, the simple slope method is used to analyze the moderating effect of health motivation on the framing effect (Figure 2). The results revealed that when consumers’ health motivation was insufficient, the mean value of consumers’ intentions to purchase artificial meat under the negative information frame and positive information frame were M _negative information frame_ = 3.14, and M _positive information frame_ = 3.33; when consumers’ health motivation was stronger, the mean values of consumers’ intention to purchase under the negative information frame and positive information frame were M _negative information frame_ = 3.34, and M _positive information frame_ = 3.65. The above data show that consumers with insufficient health motives have lower purchase intentions under the two information frameworks than consumers with stronger health motives. Moreover, consumers with insufficient health motives have fewer changes in their purchase intentions under the two information frameworks than consumers with stronger health motives, which shows that they are not easily affected by the framing effect. Possible reasons for this are that consumers with low health motivation have lower levels of dietary health concerns, lower levels of perceived health risks that may result from chronic excessive consumption of livestock meat, and lower levels of perceived benefits of consuming artificial meat. Therefore, this group of consumers is limitedly influenced by framing information, and their purchase intention is low. Meanwhile, consumers with stronger health motivation are more concerned about dietary health and can perceive the health benefits of consuming artificial meat under the positive information framework. Therefore, the purchase intention of this group of consumers is higher under the positive information frame than under the negative frame.

**Table 5:** Moderating effect of health motivation on frame effect.

Variables	df	Mean Squares	*F*	*p*-value
Message frame (M)	1	88.106	90.641	0.000
Health motivation (H)	1	94.487	97.205	0.000
M^*^H	1	4.861	5.000	0.025

## Conclusion

Based on the framing effect theory, this study investigated the effects of different types of information framing on consumers’ purchase intention of artificial meat and further analyzed the moderating effects of consumers’ knowledge level and health motivation on the framing effect. The results showed that the framing effect significantly affected consumers’ purchase intentions for artificial meat. Moreover, consumers are more willing to buy artificial meat in the context of positive information. In addition, consumers’ knowledge level and health motivation significantly moderated the influence of the framing effect on purchase intention. Consumers with more knowledge about artificial meat were less affected by the framing effect, and consumers with less health motivation were less affected by the framing effect.

The above conclusions can provide a some references for improving consumers’ purchase intention to plant-based meat products. First, the government and related companies should make full use of various news media, such as the Internet, television, radio, and newspapers, to strengthen the popularization of artificial meat knowledge, improve consumers’ knowledge level, and focus on promoting positive and positive information about artificial meat to consumers. First, the government and relevant enterprises should make full use of the Internet, television, and radio, newspapers, and other news media, strengthen artificial meat knowledge of popular science propaganda, improve the level of consumer knowledge, popularize artificial meat to consumers the benefits to human health and environmental protection, guide consumers to form scientific and correct artificial meat cognition, to enhance consumers’ purchase intentions. Second, to take advantage of the opportunity of the State Council to implement the “Healthy China Action (2019–2030),” the government and relevant departments should enhance propaganda of healthy diet through the government, society, and individual tripartite coordination, spread scientific knowledge, dietary guide consumers to form healthy, safe food concepts, improve their attention on a healthy diet. Besides, establish and improve the food safety certification system of artificial meat to improve the level of consumer trust (Chen et al., 2019).

Owing to the limited space in the questionnaire, this study only examined the influence of the information frame on consumers’ purchase intention of artificial meat and the moderating effect of knowledge level and health motivation on the information frame. This study does not explore the influence of consumer personality traits on the framework effect. In the follow-up research, we aim to focus on the role of consumer personality traits in the framing effect on consumers’ purchasing intention of artificial meat.

## Data Availability Statement

The raw data supporting the conclusions of this article will be made available by the authors, without undue reservation.

## Ethics Statement

The studies involving human participants were reviewed and approved by Jiangnan University. The patients/participants provided their written informed consent to participate in this study.

## Author Contributions

LS: validation. LS and XJ: writing—original draft. XJ: data curation and formal analysis. LW, LX, and YS: conceptualization and writing—review and editing. All authors contributed to the article and approved the submitted version.

## Funding

This work was supported by Major Project Sponsored by the National Social Science Fund of China: Research on social co-governance of food safety risks and cross-border cooperative governance mechanism (20&ZD117).

## Conflict of Interest

The authors declare that the research was conducted in the absence of any commercial or financial relationships that could be construed as a potential conflict of interest.

## Publisher’s Note

All claims expressed in this article are solely those of the authors and do not necessarily represent those of their affiliated organizations, or those of the publisher, the editors and the reviewers. Any product that may be evaluated in this article, or claim that may be made by its manufacturer, is not guaranteed or endorsed by the publisher.
